# Supplementary material for: RNA polymerase II is recruited to DNA double-strand breaks for dilncRNA transcription in Drosophila
Source: RNA Biol. 2021 Dec 29;19(1):68–77. doi: 10.1080/15476286.2021.2014694 (PMC8786327; doi:10.1080/15476286.2021.2014694)

**Supplementary Figure legends**

**Figure S1:** Outline of the NET-seq procedure and sample gel of quality control for cleavage efficiency

**Figure S2:** Genome-wide enrichment analysis for RNA polymerase II and III

A) Mapping all reads to the precompiled protein-coding sequences (= ATG-to-stop) reveals selective enrichment upon IP of RNA polymerase II

B) Mapping all reads to the precompiled intron sequences reveals enrichment upon IP of RNA polymerase II in many cases, but a number of introns appear to be at least in part transcribed by RNA polymerase III. Manual inspection of a subset indicates that these often harbor highly abundant non-coding RNA genes (snRNAs, snoRNAs etc.); it is unclear whether this shows *bona fide* pol-III transcription or contamination by abundant RNA species.

C) At least a subset of tRNA genes is clearly enriched upon IP of RNA polymerase III.

D) Mapping all reads to the precompiled “all transcripts” collection (= protein-coding and non-coding RNA polymerase II transcripts) reveals selective enrichment upon IP of RNA polymerase II with the notable exception of the *Ntl* locus.

The position of the *Act5C* transcript shown in Figure 1A of the manuscript is highlighted in panels A, B and D.

E) The *Ntl* locus harbors a Tyr-GTA tRNA gene in the first intron, the mapping traces demonstrate that the assigned reads only come from the tRNA gene and that they are clearly enriched in RNA polymerase III NET-seq libraries (tracks scaled according to total genome matching reads in each library). Please note that there are 6 Tyr-GTA tRNA genes in the fly genome with identical mature sequence and only the gene within the *Ntl* locus contains an intron. Because of the identical sequence, transcripts arising from any of the 6 tRNA loci will be mapped to each of the 6 genes. We thus cannot conclude that the *Ntl* locus, which fortuitously harbors a tRNA gene, is pol-III transcribed, but only that at least one of the Tyr-GTA tRNA loci is pol-III transcribed.

**Figure S3:** Quantification of NET-seq and input material for a set of genes (*CG15098*, *Tctp*, *CG15099* and *Act5C*).

**Figure S4:** Dose-response measurements for RNA polymerase III inhibitor treatment (ML60218). The drug was added to the cells and two hours later a mix of *Renilla* and firefly luciferase expression plasmids was transfected. Three days later, the cells were lysed and the firefly luciferase activity was recorded. This reports on both, efficient transfection and cell viability. An inhibitor concentration of 10 µM was chosen for our experiments, since it was the highest inhibitor concentration that still produced acceptable signal levels for the dual luciferase experiment (*Renilla* luciferase is the reporter, firefly is used for normalization). In mammalian cells, a concentration of 40 µM is commonly used, but in our *Drosophila* cells this is lethal after three days.

**Figure S5:** We cloned a fragment of the *CG15098* second intron downstream of the RNA pol-III promoter for the U6 snRNA (pRB17) in both sense and antisense orientation. The plasmids were then transfected into S2-cells (50 ng, mixed with 200 ng of GFP expression plasmid pKF63) and 4 days later total RNA was isolated, contaminating DNA was digested with DNase-I and the RNA was reverse transcribed using random hexamers as primers. We then performed RT-PCR reactions (35 cycles) with a primer pair placed before the A_8_ / T_8_ sequence to detect the initiated transcripts and one primer pair that spans the insertion, thus reporting on the amount of transcript that is NOT terminated across the inserted intron fragment. In three out of three replicates, the amount of non-terminated transcript was severely reduced if the intron fragment is present in the antisense orientation, i.e. comprising the T_8_ termination sequence.

The RT-PCR reporting on the amount of initiated transcript has one orientation-specific primer (necessary because of the limited sequence between transcription initiation and insertion of the intron fragment), in the case of the sense orientation some laddering has occurred for the sense orientation (A_8_) in two out of three replicates; most likely, this represents a PCR artifact. No bands were observed if the non-corresponding plasmid or the original pRB17 plasmid without any insert had been transfected, demonstrating the specificity of our amplification. We did not observe any PCR products in the corresponding minus RT samples, confirming that the signals are not due to DNA contaminations (not shown).

**Oligonucleotide sequences:**

Cloning of intron fragment:

oligo_insert_CG15098intron_pol3ter_sense

5’‑CATGGttatatgtaatctataaggcgcgcattctgt**aaaaaaaa**ccggcaagtttctttgcaaatacatatgtacatacaccatactaactagagC-3’

oligo_insert_CG15098intron_pol3ter_as 5’‑CATGGctctagttagtatggtgtatgtacatatgtatttgcaaagaaacttgccgg**tttttttt**acagaatgcgcgccttatagattacatataaC-3’

(these were phosphorylated, annealed and cloned into the *Nco*I-site of pRB17)

RT-PCR analysis:

universal sense 5’-ttaatacgactcactatatctttctaga3’

non-termianted antisense 5’-acggttcctgatgaggtggttag-3’

A8-orientation antisense 5’-gcgcgccttatagattacatataac-3’

T8 orientation antisense 5’-atatgtacatacaccatactaactagag-3’

**
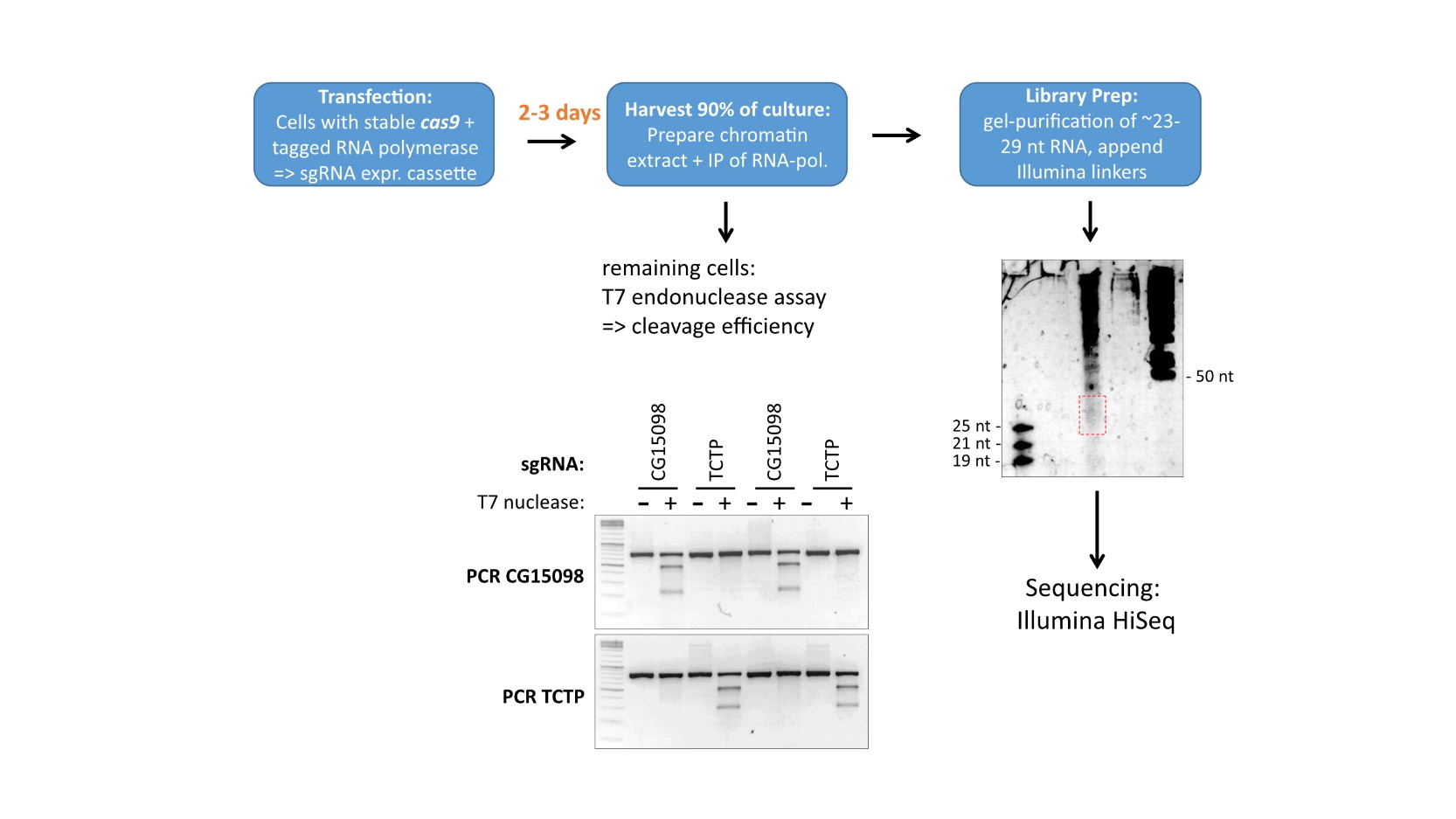
Figure S1:**

**
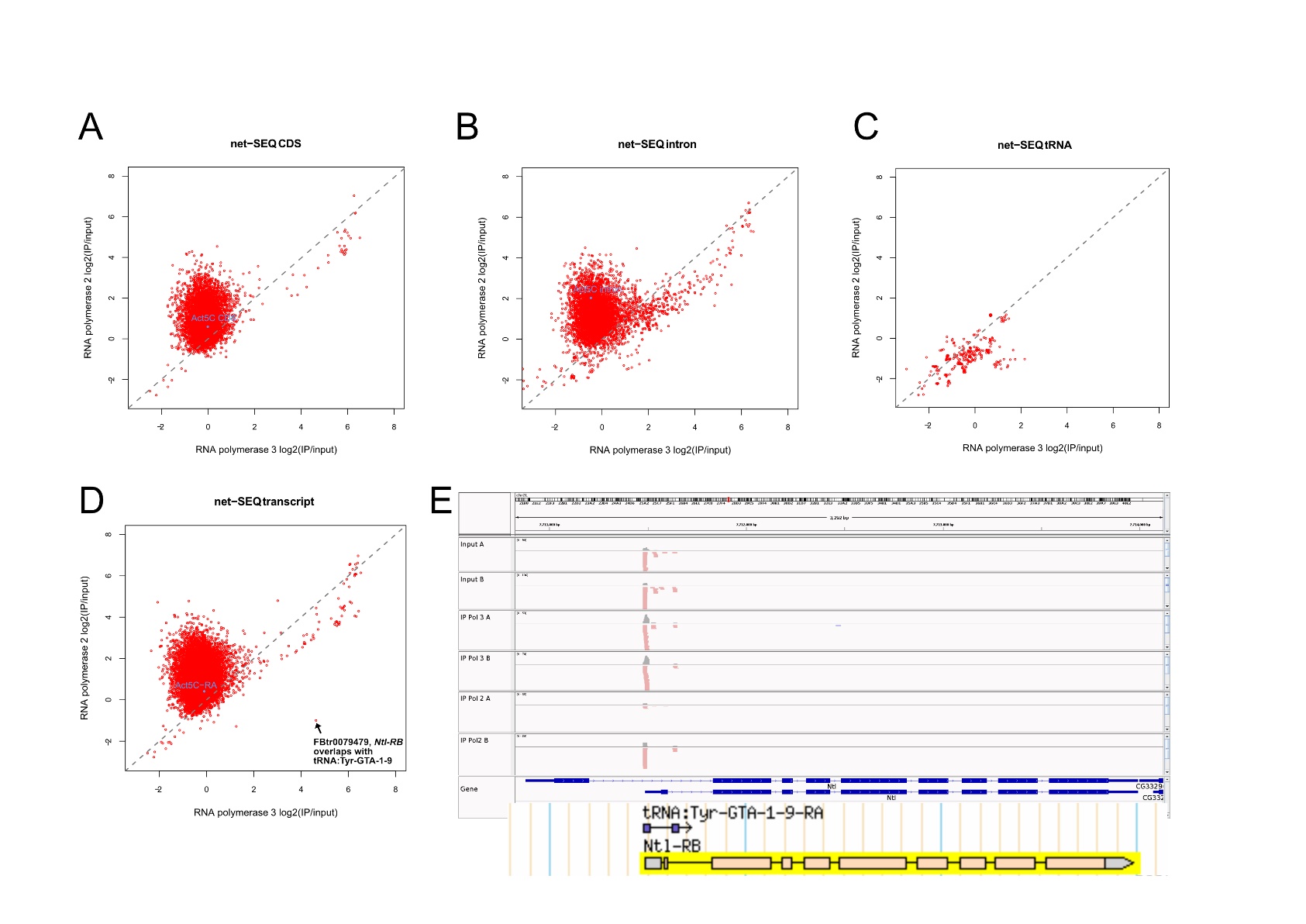
Figure S2:**

**
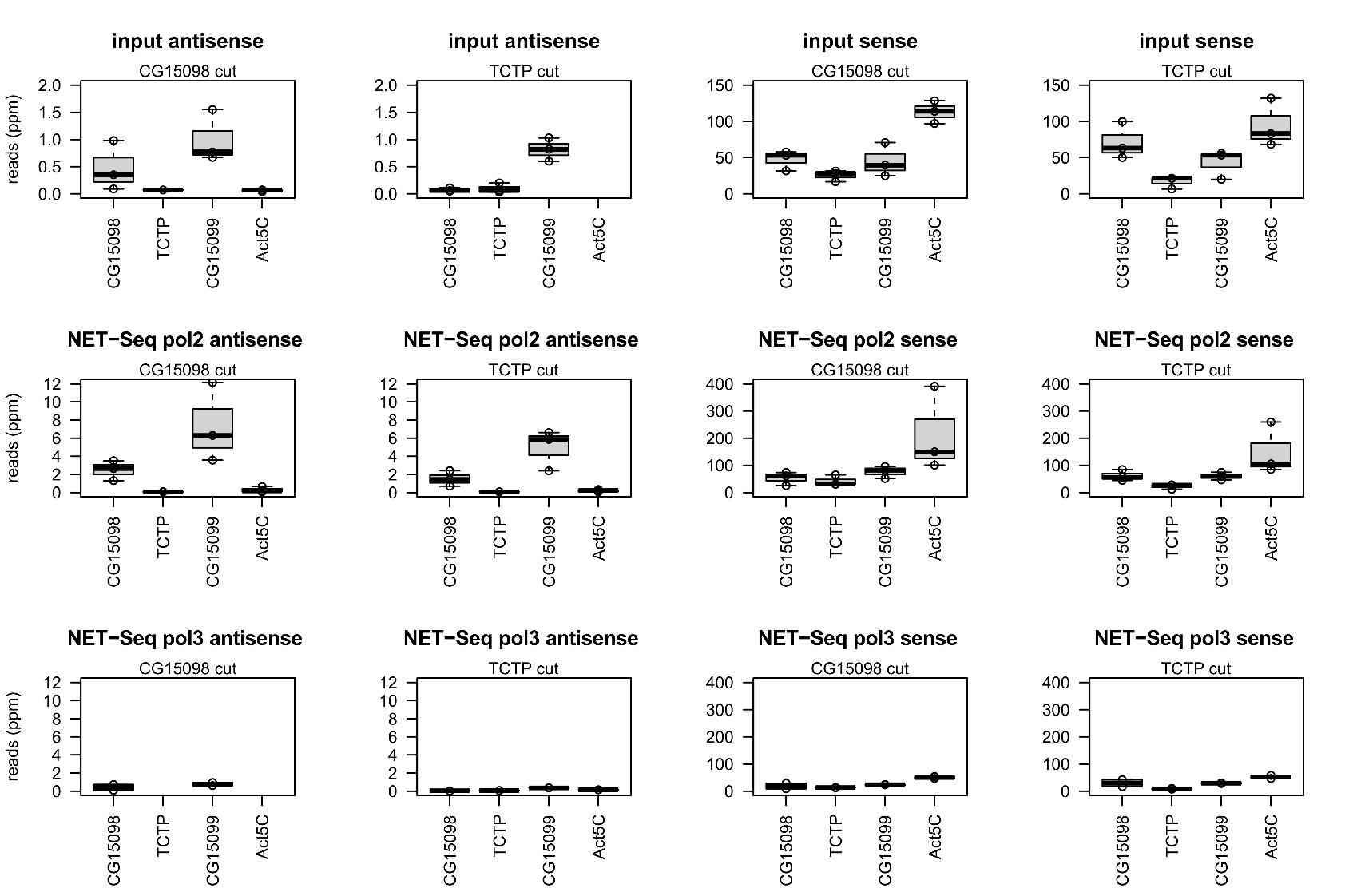
Figure S3:**

**Figure S4:**


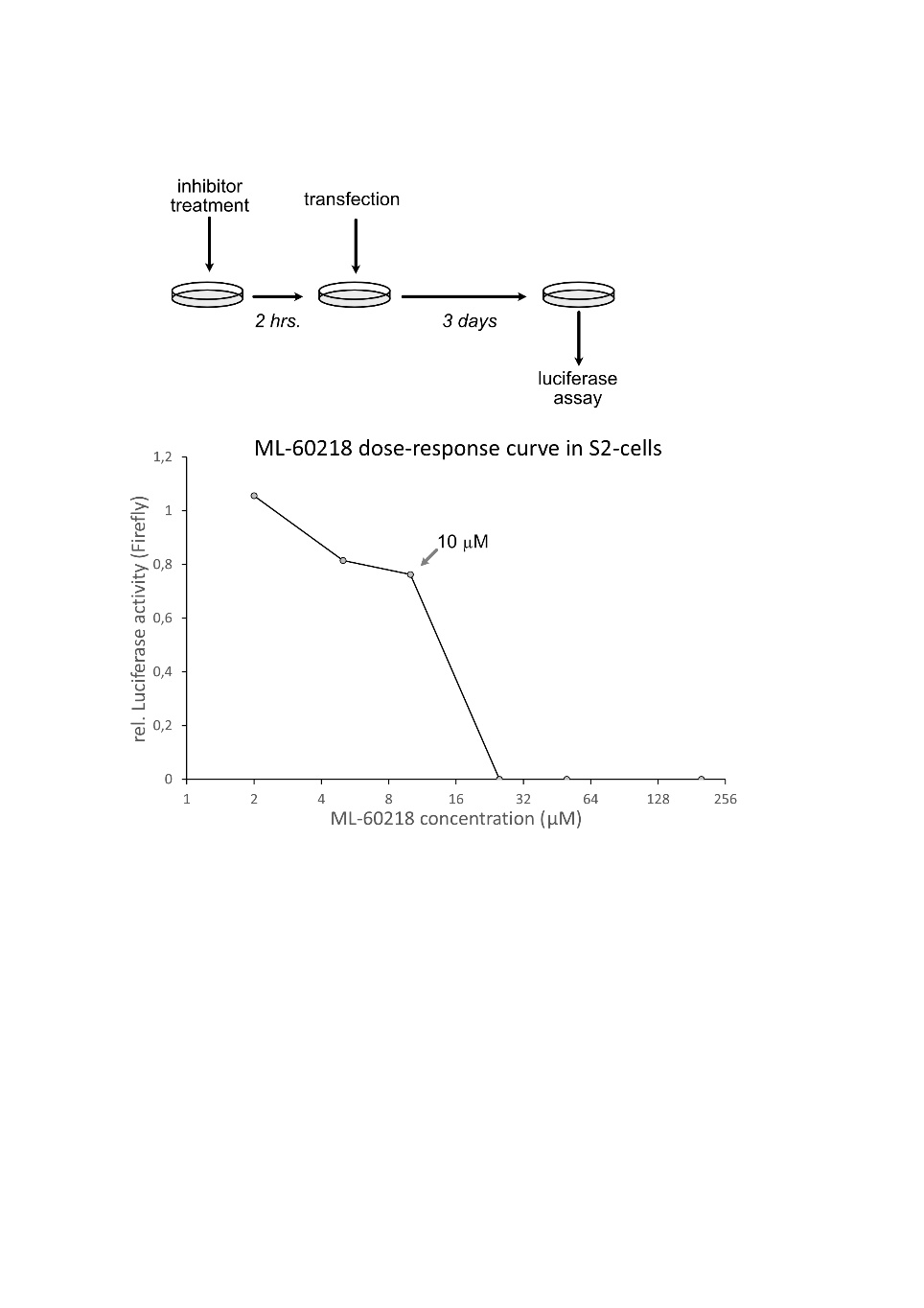


**Figure S5:**


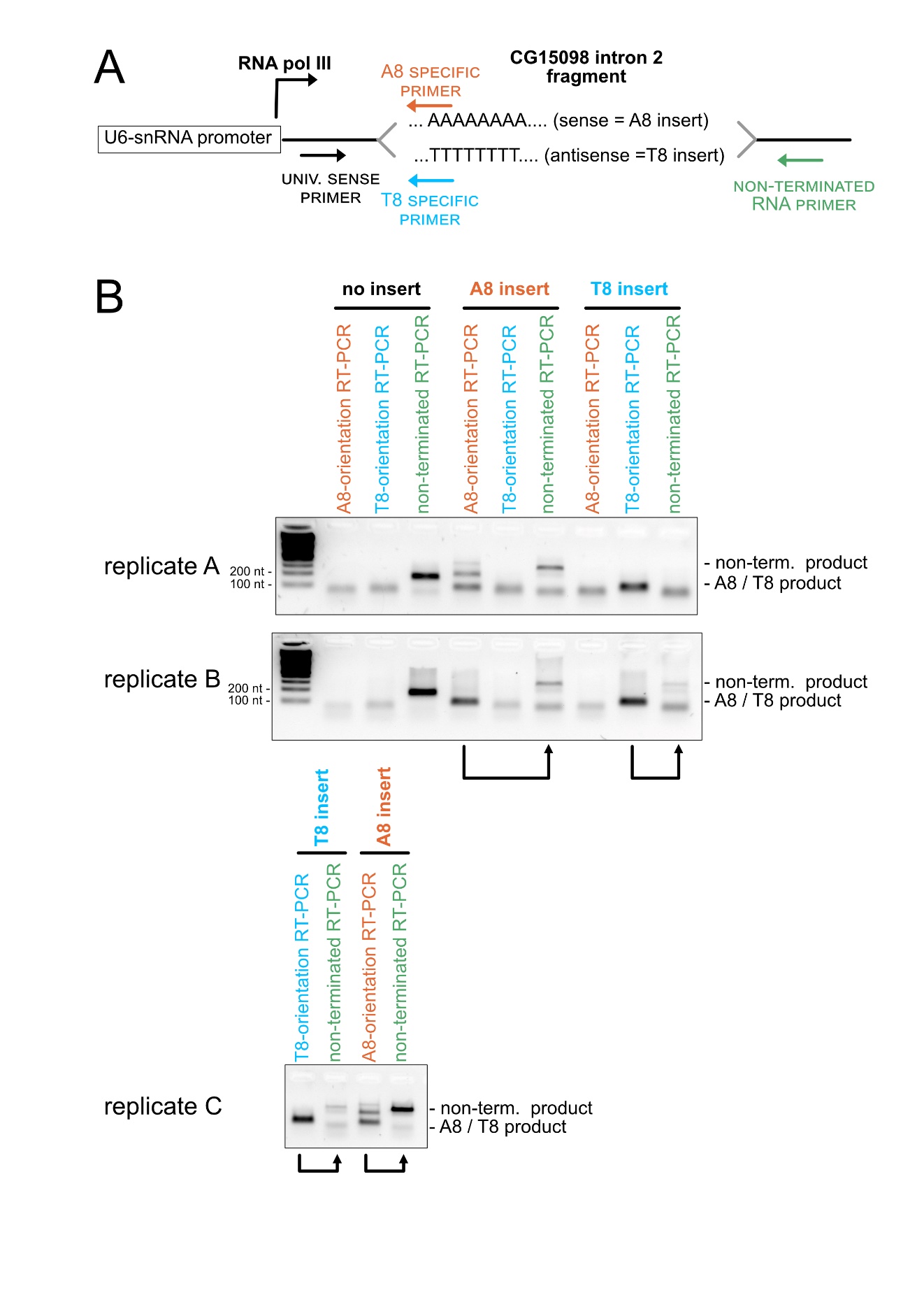

Supplement: Supplemental Material [file KRNB_A_2014694_SM9327.zip › supplementary/Supplement_Foerstemann_211129.docx]
